# Homoplastic single nucleotide polymorphisms contributed to phenotypic diversity in *Mycobacterium tuberculosis*

**DOI:** 10.1038/s41598-020-64895-4

**Published:** 2020-05-15

**Authors:** Pornpen Tantivitayakul, Wuthiwat Ruangchai, Tada Juthayothin, Nat Smittipat, Areeya Disratthakit, Surakameth Mahasirimongkol, Wasna Viratyosin, Katsushi Tokunaga, Prasit Palittapongarnpim

**Affiliations:** 1 Department of Oral Microbiology, Faculty of Dentistry, 6 Yothi Road, Mahidol University, Bangkok, Thailand; 2 Pornchai Matangkasombut Center for Microbial Genomics, Department of Microbiology, Faculty of Science, Mahidol University, Rama 6 Road, Bangkok, Thailand; 3 National Centre for Genetic Engineering and Biotechnology, National Science and Technology Development Agency, Phaholyothin Road, Pathumthani, Thailand; 4 Department of Medical Sciences, Ministry of Public Health, Tiwanon Road, Nonthaburi, Thailand; 5 Department of Human Genetics, Graduate School of Medicine, the University of Tokyo, Tokyo, Japan

**Keywords:** Genetics, Microbiology, Molecular biology, Medical research

## Abstract

Homoplastic mutations are mutations independently occurring in different clades of an organism. The homoplastic changes may be a result of convergence evolution due to selective pressures. Reports on the analysis of homoplastic mutations in *Mycobacterium tuberculosis* have been limited. Here we characterized the distribution of homoplastic single nucleotide polymorphisms (SNPs) among genomes of 1,170 clinical *M. tuberculosis* isolates. They were present in all functional categories of genes, with *pe*/*ppe* gene family having the highest ratio of homoplastic SNPs compared to the total SNPs identified in the same functional category. Among the *pe/ppe* genes, the homoplastic SNPs were common in a relatively small number of homologous genes, including *ppe18*, the protein of which is a component of a promising candidate vaccine, M72/AS01E. The homoplastic SNPs in *ppe18* were particularly common among *M. tuberculosis* Lineage 1 isolates, suggesting the need for caution in extrapolating the results of the vaccine trial to the population where L1 is endemic in Asia. As expected, homoplastic SNPs strongly associated with drug resistance. Most of these mutations are already well known. However, a number of novel mutations associated with streptomycin resistance were identified, which warrants further investigation. A SNP in the intergenic region upstream of *Rv0079* (*DATIN*) was experimentally shown to increase transcriptional activity of the downstream gene, suggesting that intergenic homoplastic SNPs should have effects on the physiology of the bacterial cells. Our study highlights the potential of homoplastic mutations to produce phenotypic changes. Under selective pressure and during interaction with the host, homoplastic mutations may confer advantages to *M. tuberculosis* and deserve further characterization.

## Introduction

*Mycobacterium tuberculosis* (Mtb) is a successful human-adapted species that has undergone long-term coevolution with its human host^1^. Previous studies^2,3^ revealed the clonal expansion of Mtb, indicating a very low possibility of horizontal gene transfer between strains. Therefore, genetic variations in Mtb appear to be primarily derived from nucleotide substitutions, insertions, and deletions^4,5^. The global population of *M. tuberculosis* sensu stricto is grouped into five phylogenetic lineages, including L1 (Indo-Oceanic family); L2 (East Asian family); L3 (East-African Indian family); L4 (Euro-American); and L7^6,7^.

In the past decade, several studies have demonstrated associations between Mtb lineages with demographic profiles, geographic distribution, transmission capacity, pathogenesis, cytokine induction, and drug resistance^6,8–10^.Once a strong association has been established, it may be explained through the identification of genotype-specific mutations, in coding or intergenic regions^11–13^. Some phenotypes, such as drug resistance, usually occur in several phylogenetic lineages. Mutations that promote drug resistance can be identified by correlating the resistance phenotypes with convergent mutations or homoplastic single nucleotide polymorphisms (SNPs). These mutations in the Mtb isolates would be expected to enhance survival in the presence of anti-TB drugs^14–18^. In addition, associations have been found between homoplastic mutations with increased Mtb transmission rate^19^ and TB disease phenotypes, such as tuberculous meningitis^20^. The studies primarily examined homoplastic SNPs in coding regions and did not examine the distribution of the homoplastic SNPs in relation to the genotypes. In most cases, when identified, intergenic homoplastic SNPs have not been experimentally evaluated.

We previously sequenced 1,170 Mtb isolates and characterized the sublineage-specific SNPs. Our analysis demonstrated correlations of Mtb genotypes with ages, ethnicity, HIV infection, and drug resistance^21^. All isolates were obtained from the same geographic area and health service system, and likely to be share some selective pressures. Here, we characterized the distribution of homoplastic SNPs in both intergenic and coding regions of the Mtb genomes that were identified in five isolates or more. This analysis provided some clues on the functions of the genes and their relevance to Mtb survival in the host environment. Furthermore, the functional effect of a homoplastic mutation in a regulatory region was experimentally examined in this study.

## Results

### Distribution of homoplastic SNPs in *M. tuberculosis* genome

The homoplastic SNPs were identified in 1,170 clinical isolates, with 480, 521, 11 and 158 isolates belonging to L1 to L4, respectively. 1,229 homoplastic SNPs were identified in 5 isolates or more. 1,121 SNPs (91%) were in the coding sequences of 589 annotated genes and 108 SNPs in intergenic regions (Table 1 and Supplementary Table S1).

**Figure 1:**
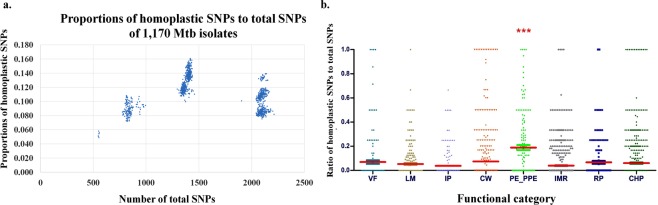
(**a**) The plots between the ratios of the numbers of homoplastic SNPs per total SNPs and the total SNPs of the 1,170 Mtb isolates. The ratios of homoplastic SNPs per total SNPs were markedly different between lineages so that the major groups from left to right are L4 (0.089 ± 0.011), L2 (0.130 ± 0.013), L3 (0.110 ± 0.006), and L1 (0.099 ± 0.015), respectively. The small leftmost group represents L4.8 which is most closely related to H37Rv, a member of L4.9. (**b**) The ratio of the number of homoplastic SNPs to total SNPs of each Mtb gene categorized into 8 functional categories: *i)* virulence and detoxification (VF, 226 genes), *ii)* lipid metabolism (LM, 238 genes), *iii)* information pathway (IP, 232 genes), *iv)* cell wall and cell process (CW, 751 genes), *v) pe/ppe* family protein (PE_PPE, 168 genes), *vi)* intermediary metabolism and respiration (IMR, 898 genes), *vii)* regulatory protein (RP, 193 genes), *viii)* conserved hypothetical protein (CHP, 1,163 genes). Each dot represents a gene that harbored at least a SNP. Statistical differences were evaluated with the nonparametric Kruskal-Wallis test. Asterisks (***) indicate the statistical significance (*p* < 0.001).

As we used the genomes of the H37Rv strain, belonging to L4.9, as the reference for identifying SNPs, the numbers of total identified SNPs would depend on the lineages of the studied isolates, which might also correlate with the numbers of homoplastic SNPs. Indeed, L1, the most distant lineage from L4, harbored the most numbers of total SNPs as well as homoplastic SNPs, with the average of 207 homoplastic SNPs per isolate compared to the average of 74 for L4 and 180 for L2. However, the average ratio of homoplastic SNPs to total SNPs of Mtb L2 isolates was 0.13, significantly higher than other Mtb lineages (One way ANOVA, Kruskal Wallis test, *p* < 0.001) (Fig. 1a), which were 0.099 for L1, 0.11 for L3 and 0.089 for L4.

We further compared the average ratios of homoplastic SNPs per total SNPs in 8 different functional categories of the genes, as shown in Fig. 1b. The *pe/ppe* genes had a significantly higher average ratio than those of the others (One way ANOVA, Kruskal Wallis test, *p* < 0.001). This is consistent with the high density of homoplastic SNPs (number of SNPs per kb) of *pe/ppe* genes, which was significantly higher than that of information pathway, intermediary metabolism, and lipid metabolism with *p*-value <0.01 (Table 1). Next, we compared the number of homoplastic SNPs in the genes of each functional group in 4 different Mtb lineages. The average ratio of homoplastic SNP to total SNPs of *pe/ppe* family of L4 was significantly lower than those of L1, L2 and L3 (One way ANOVA, Kruskal Wallis test, *p* < 0.001) (Supplementary Figure S1).

More than one-third of the identified homoplastic SNPs occurred in the *pe*/*ppe* gene family, with 96% in the coding regions as shown in Table 1. However, among the *pe*/*ppe* gene family, the distribution of SNPs in coding sequences was uneven with 90 (54%) *pe/ppe* genes harboring no homoplastic SNPs in the studied population. In contrast the homoplastic nonsynonymous SNPs arose to a density of more than 0.5% of nucleotides in 5 *ppe* genes, namely *ppe18, ppe19, ppe57, ppe59* and *ppe60*, as shown in Supplementary Table S2, indicating a high level of genetic variation. It should be noted that *ppe18, ppe19* and *ppe60* were highly homologous while *ppe57, ppe58* and *ppe59* were a separate homologous group^22^. *ppe57*, *ppe59*, and *ppe60* were previously reported to be highly polymorphic^23^. Compared to the other gene categories, the percentage of *pe*/*ppe* genes containing the homoplastic SNPs in the regulatory region was also high (6% of total *pe*/*ppe* genes) (Table 1).

**Table 1:** Distribution of intergenic homoplastic SNPs and homoplastic nonsynonymous SNPs in eight different gene categories.

Functional Category (No. of genes)	No. of 5’ upstream regions carrying homoplastic SNPs (%)	No of coding sequences carrying homoplastic SNPs (%)	Ratio between No. of upstream regions and coding sequences having homoplastic SNPs	No. of homoplastic SNPs in 5’ upstream regions	No. of homoplastic SNPs in coding sequences	Ratio between No. homoplastic SNPs in upstream and in coding sequences	No. of homoplastic SNPs in coding regions		Ratio of nsSNP/sSNP	No. homoplastic SNPs per gene length (per kb)^ɸ^		No. homoplastic nsSNPs per gene length (per kb)	
							nsSNP	sSNP		Median	Min-Max	Median	Min-Max
Cell wall and cell process (751)	15 (2.0%)	111 (14.8%)	0.14	18	199	0.09	112	87	1.28	1.11^$^	0.2–59.65	0.65	0.00–38.60
Conserved hypothetical (1,163)	22 (1.9%)	143 (12.3%)	0.15	26	174	0.15	114	60	1.90	1.20^*^	0.29–12.20	0.82	0.00–5.95
Information pathway (232)	6 (2.6%)	29 (12.5%)	0.21	6	33	0.18	21	12	1.75	0.59	0.30–5.38	0.40	0.00–5.38
Intermediary metabolism and respiration (898)	15 (1.7%)	115 (12.8%)	0.13	17	133	0.13	75	58	1.29	0.85	0.27–3.66	0.56	0.00–3.55
Lipid metabolism (238)	7 (2.9%)	52 (21.8%)	0.14	7	76	0.09	40	36	1.11	0.62	0.15–2.19	0.38	0.00–1.52
*pe*/*ppe* (168)	10 (6.0%)	78 (46.4%)	0.13	19	421	0.05	212	209	1.01	1.28^*^	0.15–31.46	0.72	0.00–18.71
Regulatory proteins (193)	9 (4.7%)	34 (17.6%)	0.27	12	36	0.33	24	12	2.00	1.18	0.31–2.85	0.77	0.00–2.85
Virulence, detoxification, adaptation (226)	3 (1.3%)	27 (11.9%)	0.11	3	49	0.06	29	20	1.45	0.93^$^	0.25–27.97	0.78	0.00–13.99
**All categories (3,869)**	**87 (2.2%)**	**589 (15.2%)**	**0.15**	**108**	**1,121**	**0.096**	**627**	**494**	**1.27**	**0.99**	**0.15–59.65**	**0.65**	**0.00–38.60**

^*^Total SNP densities of conserved hypothetical proteins and *pe/ppe* were significantly higher than those of information pathway, intermediary metabolism and respiration and lipid metabolism (One way ANOVA, Kruskal Wallis test *p*-value <0.01).

^**$**^Total SNP densities of cell wall/cell process and virulence, detoxification were significantly higher than those of lipid metabolism (One way ANOVA, Kruskal Wallis test *p*-value <0.001 and <0.05, respectively).

^**ɸ**^The number of homoplastic SNPs per gene length (per kb) was calculated only for genes carrying homoplastic SNPs.

Homoplastic SNPs were not distributed evenly in all 4 Mtb lineages, as shown in Supplementary Table S3. 40% of the homoplastic SNPs were identified only in a single lineage. These SNPs could be identified only when the bacterial samples were classified into sublineages. 10% of the homoplastic SNPs were distributed in all 4 Mtb lineages (Supplementary Table S3).We identified some SNPs in all studied isolates except the ones belonging to the same clades as the H37Rv strain or L4.9. The clades could be L4.8 and L4.9 or L4.5, L4.8 and L4.9 or L4.3, L4.4, L4.5, L4.8 and L4.9. The SNPs, which were absent in these monophyletic groups, were not considered as homoplastic SNPs. Approximately 30% of the homoplastic SNPs occurred in more than 100 clinical *M. tuberculosis* isolates (Supplementary Figure S2).

In addition to the functional classification, the genes of *M. tuberculosis* were also classified based on whether they were essential, contributed to virulence, or exhibited antigenicity. We compared the density of nsSNPs per kb in each category (Supplementary Table S4). The homoplastic nsSNPs were more common in non-essential than essential genes. Nevertheless, the frequencies of nsSNPs identified in virulence and antigenic protein genes were not statistically different from non-virulence and non-antigenic genes, respectively. 57 (25%) of the 226 homoplastic nonsynonymous SNPs in antigenic protein genes caused missense mutations in T cell epitopes as shown in Supplementary Table S5, with 53 (93%) of them being in *pe*/*ppe* family and ESX family proteins.

PPE proteins share a relatively conserved N-terminal domain with a proline-proline-glutamic acid motif. Interestingly, the N-terminal regions of PPE18, PPE57, and PPE60 proteins interact with the TLR2 receptor present in macrophages^24–26^ and modify the host responses by inducing IFN-γ production^25,27,28^. These genes are candidates for TB vaccine development. To explore more on the possible cause of genetic variations among the 5 *ppe* genes, we calculated dN/dS values of the whole genes, their T cell epitope regions and non-T cell epitope regions as shown in Table 2. The dN/dS of T cell epitopes of the five *ppe genes* were generally less than one, and usually less than the non-T cell epitopes, suggesting purifying selection, even though the density of homoplastic SNPs was high. The only exception is the dN/dS of the T cell epitopes of *ppe57*, of L2 isolates which was more than one, suggesting positive selection (Table 2).

**Table 2:** dN/dS of the whole genes, T cell epitope regions and non-T cell epitope regions of 5 *ppe* genes with the highest homoplastic SNP density.

Gene	All Lineages			Lineage1 (n = 480)			Lineage2 (n = 521)			Lineage3 (n = 11)			Lineage4 (n = 158)		
	Whole region	T cell epitope	non T cell epitope	Whole region	T cell epitope	non T cell epitope	Whole region	T cell epitope	non T cell epitope	Whole region	T cell epitope	non T cell epitope	Whole region	T cell epitope	non T cell epitope
*Rv1196 (ppe18)*	0.76	0.73	0.79	0.80	0.80	0.63	0.63	0.41	0.99	0.74	0.27	**2.18**	0.52	0.45	0.65
Rv1361c* (ppe19)	0.68	0.68	—	0.64	0.64	—	0.36	0.36	—	0.25	0.25	—	0.46	0.46	—
Rv3425* (ppe57)	0.60	0.60	—	0.38	0.38	—	**1.31**	**1.31**	—	0.38	0.38	—	0.76	0.76	—
*Rv3429*^*$*^ *(ppe59)*	**1.80**	0.00	**1.79**	**2.14**	0.00	**2.13**	**1.40**	0.00	**1.38**	0.00	0.00	0.00	**1.22**	0.00	**1.20**
*Rv3478 (ppe60)*	0.78	0.39	1.05	0.72	0.28	0.96	0.80	0.61	0.94	0.71	0.00	**1.39**	0.65	0.31	0.85

*PPE19 and PPE57 antigens had no non-T cell epitope regions so the dN/dS ratio was calculated only in the T cell epitope region.

^$^No missense mutation occurred in the T cell epitope region of PPE59.

**Figure 2:**
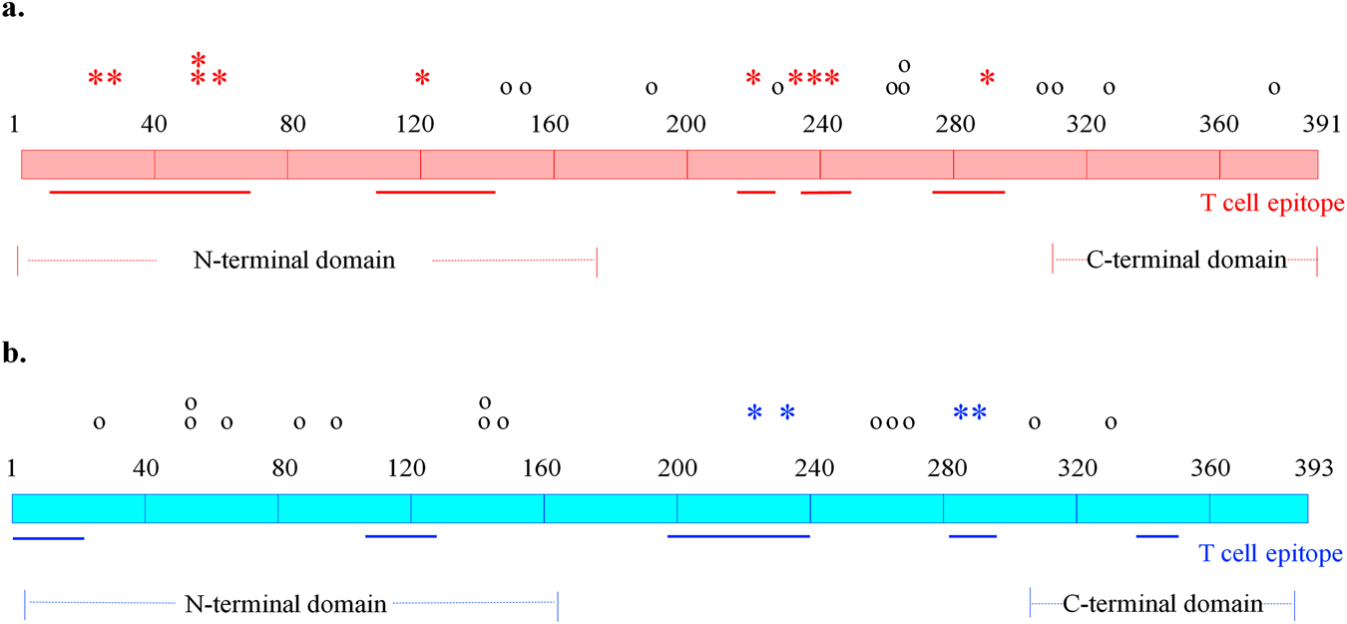
A diagram of *M. tuberculosis* PPE18 **(a)** and PPE60 **(b)** proteins. PPE18 is composed of 391 amino acids, with amino acids 1–180 classified as the N-terminal domain responsible for TLR-2 binding. The PPE60 protein is 393 amino acids in length, with amino acid residues 6–163 being the N-terminal domain, while amino acids 309–389 contained the C-terminal domain. T cell epitope regions were mapped as solid lines. The locations of homoplastic nsSNPs were shown. Asterisks indicate nsSNPs affecting the T cell epitopes and circles represent nsSNPs outside the T cell epitope regions.

**Figure 3:**
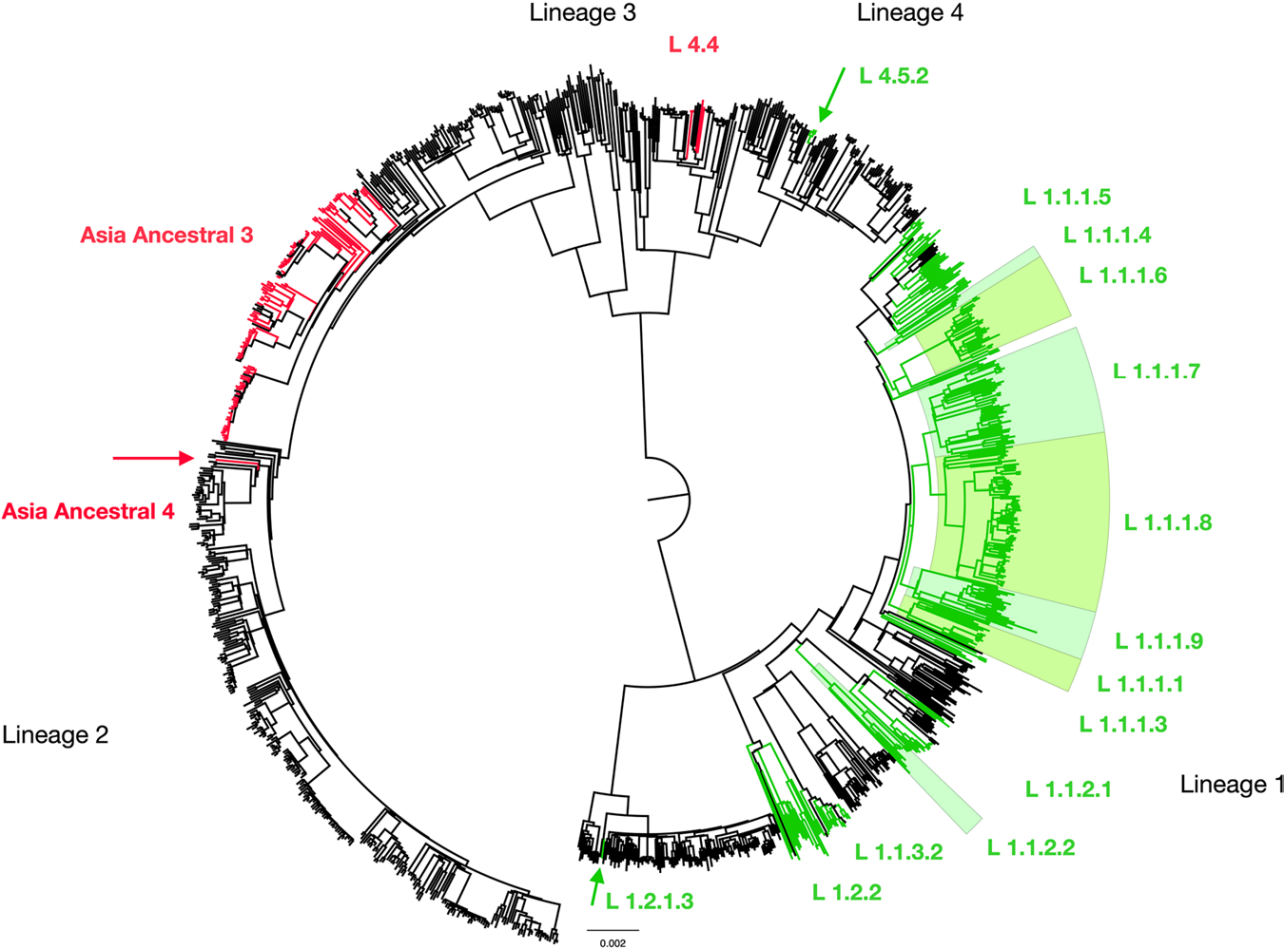
Position of homoplastic C1339436A SNP (C88A) and G1340052T SNP (G704T) of *ppe18* in phylogenetic tree of 1,170 *M. tuberculosis* isolates. The phylogeny was reconstructed using Bayesian Interference (BI) methods, which included 4 major lineages (L1: Indo-Oceanic family, L2: East Asian family, L3: East-African Indian family, L4: Euro-American) and 38 sublineages belonging to L1, L2 and L4. Red terminal lines correspond to Mtb isolates carrying homoplastic C88A, and green terminal lines represent the isolates carrying homoplastic G704T of *ppe18*. The C88A mutation mostly presents in 76% of L2.2.1 Asia Ancestral 3 isolates, 1 isolate of L2.2.1 Asia Ancestral 4 and 4 isolates of L4.4. While, the G704T SNP was found in 86% of L1 isolates and 3 isolates of L4.5.2. Color background shading represents all isolates of that sublineage carrying the homoplastic SNPs.

PPE18 is a subunit of a promising TB vaccine candidate, M72/AS01E, which has been evaluated in a phase 2b clinical study^29^. Of the 65 nucleotide variants identified in PPE18 from the 1,170 L1-L4 *M. tuberculosis* isolates shown in Supplementary Table S6, 37 SNPs were found in more than 5 isolates and 42 SNPs (65%) being non-synonymous. 11 of the 22 homoplastic nsSNPs (50%) resulted in changes in the T cell epitopes (Fig. 2a). Eight homoplastic nsSNPs were located in the relatively conserved N-terminal domain, which is involved in the interaction with TLR2 of host cells. L1 isolates harbored 20 of the 22 homoplastic nsSNPs while the L2 isolates harbored 11.Three previously reported mutations G704T, G860A, and T926C^30,31^ were commonly present. G860A, a mutation affecting a T cell epitope, was actually present in all L1 isolates and all Beijing strains (L2.2), but not the Proto-Beijing sublineage (L2.1) as shown in Supplementary Figure S4. G704T (Fig. 3) and T926C were found in 86% and 53% of L1 isolates, respectively but not L2. The presence of G860A in all L1 and the Beijing strains were also confirmed in a set of the complete genomes of 6 L1 and 35 L2 isolates deposited in Genbank (Supplementary Table S7). G704T were present in all 6 complete L1 genomes and T926C were present in 5 L1 genomes while both were not found in any complete genomes of L2. C88A, also affecting a T cell epitope, was present almost exclusively to a sublineage of the Beijing strains, L2.2.1 Asia Ancestral 3, with 76% (112/146) harboring the SNP (Fig. 3). Four L4 isolates also contained the SNP. In the complete genome set, it was present in one of the two isolates that belonged to the Asia Ancestral 3 sublineage and 23% (26/111) of the L4 isolates. Novel mutations identified in the T cell epitopes included G96C and T365C. The G96C substitution was found in 78 L1 isolates (17%), affected the same T cell epitope as C88A and was predicted to impair the function of PPE18, as shown in Supplement Table S1. The presence of two double mutations, G163A-T164C and A787C-G788A, resulted in amino acid changes, Val55Thr and Ser263His respectively.

PPE60 is a component of the ID87/GLA-SE vaccine candidate^32^, which has comparable efficacy to the BCG vaccine in mice^32^. 30 homoplastic SNPs (18 nsSNPs, 11 sSNPs, and 1 premature stop codon mutation shown in Supplementary Table S6) were found with 3 nsSNPs (16%) resulting in changes to amino acid residue sequences in T cell epitopes and 1 nsSNP leading to a premature stop codon (Fig. 2b).

### Associations between homoplastic SNPs and the demographic and clinical characteristics of the patients

The demographic and clinical characteristics of TB patients were previously reported^21^. The associations of all 1,229 homoplastic variants and various TB phenotypes including anti-TB resistance, treatment outcomes, AFB smear positivity and HIV status were evaluated (Supplementary Table S8). We found significant associations after Bonferroni correction between homoplastic SNPs with anti-TB resistance and treatment outcomes but not with HIV status and AFB smear positivity.

There were 11 homoplastic SNPs associated with the drug resistance phenotypes. Eight of them are already well known. However, associations between streptomycin resistance to mutations in *aglA* (T922C, *p* = 2.15E-13)*, pe_pgrs7* (G2353A, *p* = 4.20E-13), and *sseA* (G826A, *p* = 1.01E-12) have never been described and required further investigations. There were several SNPs associated with poor outcomes of treatment, either failure or death within a year after the start of treatment, which deserved further investigations.

### Functional characterization of a homoplastic SNP in the 5′ regulatory region of *DATIN (Rv0079)*

108 SNP loci were found in the 5′ upstream region of 87 Mtb genes. Promoters were predicted using the Neural Network Promoter Prediction program (BDGP) and Bacterial promoter prediction program BPROM. Homoplastic SNPs were predicted in 22 promoter regions with five having higher promoter scores greater than 0.8 (Supplementary Table S1). Three of the five promoters were predicted to regulate conserved hypothetical proteins with unknown functions. Only one, (−47 A/T), located 47 bp upstream the start codon of a pathogenesis-related *DATIN* (*Rv0079*) gene. *Rv0079* encodes a conserved hypothetical protein and is the only DosR regulon-encoded gene that harbors a homoplastic SNP upstream of the coding sequence. While DATIN (dormancy associated translation inhibitor) is important for the latency state of *M. tuberculosis*, the homoplastic SNP was identified in only 10 isolates, although these were distributed among all 4 lineages, as shown in Supplementary Table S1. This indicated that the mutations at this particular position likely occurred independently several times.

**Figure 4:**
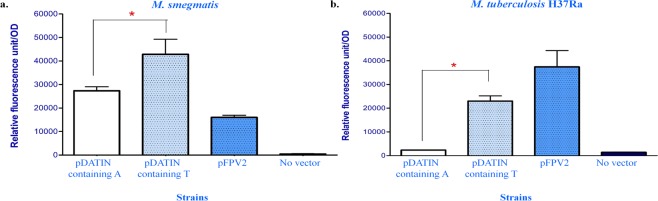
Effect of the homoplastic −47 A/T SNP on the expression of a downstream *gfp* gene. The promoter activity was expressed in relative fluorescence levels per unit of optical density (RFU; fluorescence level/OD) of GFP. The activity of promoter with T nucleotide at −47 was compared to the promoter with A nucleotide in *M. smegmatis* **(a)** and *M. tuberculosis* H37Ra **(b)**. Mycobacterial cells carrying the pFPV2 plasmid served as a positive control for GFP expression, whereas cells without the plasmid served as the negative control. The presented data were averaged RFU from three independent experiments. Asterisks represent significant difference; *p*-value <0.001.

There is no reliable computational method to predict the effect of the −47 A/T mutation in *Rv0079*. To evaluate the effect of the mutation on expression of *Rv0079*, we constructed a promoter fusion with a downstream *gfp* gene. The 150 bp-long DNA segment upstream of the start codon of *DATIN* displayed promoter activity as expected in *M. smegmatis* and *M. tuberculosis* H37Ra. Plasmids containing T at the −47 position exhibited significantly higher GFP fluorescence levels than plasmids containing A at the same position, *p*-value <0.001 (Fig. 4). Remarkably, GFP fluorescence was 10 times greater in *M. tuberculosis* H37Ra transformed with the promoter-*gfp* reporter plasmid containing T at −47 compared to the reporter with A at this position (mean RFU = 23,031 and 2,300 units, respectively). Thus the homoplastic A/T SNP in *Rv0079* regulatory region enhanced promoter activity of a downstream gene. It is quite interesting to prove whether this homoplastic SNP would have effect on level of DATIN activity in clinical isolates.

### Identification of the transcriptional start site (TSS) in *Rv0079* (*DATIN*)

**Figure 5:**
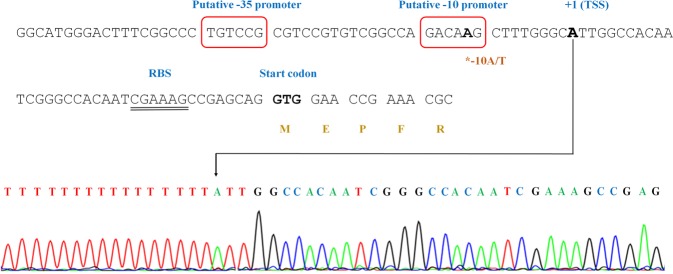
Characterization of the regulatory sequence upstream of *Rv0079* (*DATIN*). GTG is the start codon of *Rv0079*. The transcriptional start site (TSS) was identified at nucleotide 37 upstream the start codon (arrow), with the RACE chromatogram shown below. The predicted −10 and −35 promoter sequences were indicated (boxed). The double-underlined letters represent the putative ribosomal binding site (RBS) of *Rv0079*.

To establish the exact location of the −47 A/T mutation in the *Rv0079* promoter, 5' RACE was performed to determine the TSS. This analysis revealed the TSS is at the A nucleotide 37 bp upstream of the *DATIN* start codon (Fig. 5). Therefore, the homoplastic −47 A/T is 10 bases upstream of the TSS, inside the −10 promoter of *Rv0079*. The potential −35 and −10 promoter sequences are predicted to be TGTCCG-15 bp-GACAAG starting at the position −35 bases and −14 bases upstream the TSS, respectively.

## Discussion

In this work, we explored the distribution of homoplastic SNPs in a cohort of *M. tuberculosis*. The 1,170 *M. tuberculosis* isolates examined in this study were from a single province in Thailand, and were likely to be exposed to similar environments. The identified homoplastic SNPs should provide some clues on the mechanisms of survival or pathogenesis of the bacteria.

Our analysis revealed the presence of homoplastic SNPs in all categories of genes. They were less common in the essential genes, as expected. Approximately 36% of identified homoplastic SNPs were in the *pe*/*ppe* family, although more than half of the *pe/ppe* genes did not harbor any homoplastic SNPs. This suggested the diverse functions of the protein family with the more polymorphic proteins probably playing important roles in interaction with the host cells. The *pe*/*ppe* family consists of 99 *pe* and 69 *ppe* genes, accounting for 7% of total *M. tuberculosis* coding potential^22^. Many proteins in this family are cell surface-associated proteins or secreted proteins and involved in the pathogenesis of *M. tuberculosis*. The pe/ppe proteins also act as potent antigens that induce both host humoral and cell-mediated immune responses^22,33^. There are evidences that at least some *pe*/*ppe* genes are under selective pressure^23^. Our analysis was consistent with this prediction and also indicated that selective pressures were variable among different polymorphic pe/ppe proteins. Both *pe* and *ppe* genes share similar N-terminal sequences, allowing homologous recombination within and between *pe*/*ppe* genes, which may contribute to the high mutation rates observed in this gene family^34^. Recombination of *pe*/*ppe* genes may be responsible for producing a large source of antigenic variation in *M. tuberculosis*^35^ with the potential to modulate the host immune response.

The homology between members of *pe* and *ppe* gene families makes identification of SNPs in the gene family less reliable than the other groups of genes. Nucleotide variants in *pe*/*ppe* genes are therefore generally excluded from phylogenetic analysis. In order to increase the reliability of the identification of SNPs in *pe/ppe* genes, we used two SNPs identification methods and analyzed only the SNPs identified by both methods. We also analyzed the presence of common homoplastic SNPs in the complete genomes of *M. tuberculosis* deposited in Genbank, the results of which were consistent with the results from the set of 1,170 clinical isolates.

Three of the most polymorphic PPE proteins, PPE18, PPE57, and PPE60, have been shown to interact with TLR2 receptors. Many polymorphisms have been identified in TLR2^36^ with at least two mutations, R677W^37^ and R753Q^38,39^, have been known to impact tuberculosis and other mycobacterial infections. These residues are within the extracellular domain of TLR2 and may be involved in PPE-TLR2 interactions. The mutations in the *ppe* genes may provide a molecular mechanism for human-Mtb co-evolution. However, the structural consequences of the SNPs in *ppe18*, *ppe 57* and *ppe 60* remained to be determined.

There were differential associations between the *pe*/*ppe* polymorphisms and lineages. The *ppe18* was most polymorphic among L1 isolates while *ppe57* was more polymorphic among L2 isolates (Table 2 and Supplementary Table S6). The selective pressure acting on *pe*/*ppe* genes appeared to depend on sublineages, as indicated by dN/dS. For example, *ppe18* was highly polymorphic among L1 isolates, yet this gene was under purifying selective pressure in all lineages. For most lineages, the selective pressure on *ppe57* was purifying selection; however, the protein was under positive selective pressure in L2 which appeared to drive them to be more polymorphic.

PPE18 is a component of the M72/AS01E vaccine candidate, a fusion protein derived from two Mtb antigens, pepA and PPE18. Administration of the M72/AS01E vaccine to HIV-negative adults with latent TB infections had a 54% efficacy in preventing the development of active TB disease^29^. However, sequences of *ppe18* among 225 clinical isolates collected from Arkansas and Turkey displayed substantial variability, especially among isolates belonging to principal genetic group 1, which are mostly L1 and L2 strains^30^. The deviation of *ppe18* sequences from the H37Rv (L4) strain used to produce the vaccine candidate was reported to be high among three isolates belonging to the EAI family (L1)^31^. In contrast, Mortier *et al*.^40^ demonstrated high diversity in only one of the four EAI isolates. Here we showed variations in *ppe18* from all 4 major Mtb lineages, particularly L1. The numbers of nonsynonymous mutations (both homoplastic and non-homoplastic SNPs) in the isolates belonging to L1-L4 were 35, 16, 7 and 16 loci, respectively (Supplementary Table S6). Three *ppe18* mutations were common among L1 isolates with G860A affecting a T cell epitope and also common in the Beijing strains. Furthermore, homoplastic nsSNPs were found to affect promiscuous epitopes that can bind to multiple HLA alleles^41^. Examination of binding between PPE18 epitopes and various DRB1 of MHC class II indicates that the M72/AS01E vaccine may not be recognized well in people from many countries, including Thailand, Vietnam, Indonesia, and the Philippines^41^. These countries are all endemic to L1 strains with highly variable *ppe18* sequences. The co-evolution between *ppe18* and host cell antigen recognition indicates that precaution is needed when extrapolating data from the clinical trials in Africa to Southeast Asia. Vaccine epitope diversity could induce variable host immune responses and, therefore, may affect the novel TB vaccine efficacy^42,43^.

Ruesen *et al*.^20^ reported the homoplastic mutations in *Rv0218* and the absence of *Rv3433c* and *nanK* to be highly correlated with tuberculous meningitis. However, the SNPs in these genes were not found in this study, which included patients with pulmonary tuberculosis.The same study also identified homoplastic mutations in six pe/ppe proteins associated with pulmonary tuberculosis disease. Here we confirmed the presence of three homoplastic nsSNPs: A270T in *pe/pgrs*18, L111W in *pe/pgrs*19, and D236G in *pe/pgrs*26^20^.

The homoplastic SNPs can be intragenic or intergenic. Methods to predict the effect of intragenic SNPs are fairly well-developed while predicting the effects of intergenic SNPs is more difficult due to the considerable variability in the sequences of regulatory elements^44^.

In this study, we experimentally evaluated the effect of the homoplastic SNP upstream of *Rv0079* (*DATIN*) as it is the only DosR regulated gene that harbors the intergenic SNP. The genetic variations SNP substantially increased the expression of the downstream *gfp* reporter gene. The effect on promoter activity is presumably similar in native bacterial cells. The *Rv0079* (*DATIN*) gene is a member of dormancy survival regulon (DosR regulon)^45,46^ and the DATIN protein interacts with the 30S ribosome and inhibits protein synthesis in *M. tuberculosis*^47^. In addition, DATIN could interact with the TLR2 receptor and stimulate the secretion of pro-inflammatory cytokines (IFN-ℽ, TNF-α, IL-1β, and IL-8) by mononuclear cells^48^. These cytokines play significant roles in granuloma formation. Therefore, the SNP identified in the intergenic region upstream of the *Rv0079* (*DATIN*) gene may alter the cellular processes for entry into a dormant period. The association of a homoplastic substitution in *DATIN* regulatory region and adaptability of clinical Mtb to enter dormancy warrants further investigation. Although the prevalence of the SNP upstream *DATIN* was not high, this work demonstrated that screening for homoplastic SNPs could be a method for identifying function-affecting SNP in regulatory regions. To our knowledge, this is the first report demonstrating the functional effect of an intergenic homoplastic SNP.

In conclusion, this study evaluated homoplastic SNPs, which were not reported in most WGS studies of Mtb. The possible functional consequences of SNPs were examined and the information obtained may be useful for understanding the bacterial-host interaction, and developing new technology against TB^43^ such as identifying new drug targets as well as understanding the effects of vaccines.

## Materials and Methods

### Bacterial strains

1,170 Mtb isolates, analyzed by WGS, were obtained from pulmonary TB patients in Chiangrai province, Northern Thailand between 2003–2010. The isolates were previously classified into four major lineages, with 480, 521, 11 and 158 isolates belonging to L1–4, respectively. The three major Mtb lineages were further divided into 38 sub-lineages^49^.

### Genome sequencing data and Variant calling

Genome sequencing data of all *Mycobacterial* isolates obtained from our previous study^49^ were further analyzed, using two different variant callers, GATK^50^ and SAMtools^51^. The intersecting SNPs were used for further analysis. Criteria for single nucleotide variant filtering was used to meet high-confidence variants, according to the study of Faksri *et al*.^52^; mapping quality >50, base alignment quality >20, >10 reads covering each site. Insertions and deletions were excluded from this study. The SNP calling workflow was shown in Supplementary Figure S3.

### Genomic sequence data availability

Whole genome sequence data used in this study were deposited at European Nucleotide Archive (ENA) of EMBL-EBI under the accession numbers ERP006738.

### Homoplastic SNPs identification

Phylogenetic trees were previously constructed based on 70,937 SNPs by Maximum Likelihood (ML) and Bayesian Inference (BI) methods^21^. Both phylogenetic methods resulted in the same sublineage classifications, although the structure of the tree inside each sublineage might be slightly different. All identified SNPs were mapped in the phylogenetic trees. SNPs that were found only in a single sublineage were excluded from further analysis. The SNPs were considered as homoplastic if they occurred in two or more sublineages and did not form a monophyletic clade. The reference H37Rv strain belongs to L4, the SNPs present in all L1, L2 and L3 isolates but not in any of L4 isolates were considered to be specific to L4 and not considered as homoplastic SNPs. The homoplastic SNPs found in less than 5 isolates (approximately 0.5% of Mtb isolates) and SNPs present in mobile genetic elements and prophages were excluded from further analysis.

### Prediction of the functional consequences of homoplastic SNPs on protein functions and T cell epitopes

The effects of nonsynonymous mutations on protein functions were determined with the online tool, PredictSNP 1.0^53^, using three algorithms (SIFT, Polyphen-1, and SNAP). The programs reported the possible effects as N: neutral effect (no effect) and A: deleterious effect on protein function. Analysis of the mutations altering amino acid sequences in T cell epitopes was performed using the information catalogue in the Immune Epitopes Database (www.iedb.org). This database contains a large number of experimentally-proved T cell epitopes in *M. tuberculosis*^54^. The dN/dS ratio was calculated from all SNP types identified in our study and analyzed with the MEGA7 program^55^. The functional gene categories were extracted from Tuberculist^56^

### Identification of SNPs in the predicted promoter regions

Homoplastic SNPs residing within 200 bp upstream of the start codons from annotated genes were identified as intergenic SNPs. The promoter regions were predicted from the 5' non-coding sequences using the Neural Network Promoter Prediction computer program^57^ and Bacterial promoter prediction program BPROM^58^.

### SNP identification in complete genomes of *M. tuberculosis* deposited in Genbank

Complete genomes of Mtb were obtained from the National Center for Biotechnology Information (https://www.ncbi.nlm.nih.gov/genome/browse#!/prokaryotes/166/). The number of genomes were 6 for L1, 35 for L2, 2 for L3 and 111 for L4. The sublineage of each isolate was identified using previously published criteria^21^. Homologous genes to the ones in the H37Rv strains that contained homoplastic SNPs and subsequently the SNPs were identified.

### Experimental characterization the effect of the homoplastic SNP upstream of the *DATIN*

The effect of an intergenic homoplastic mutation on transcriptional activity was performed using a green fluorescence protein (GFP) reporter assay. Briefly, 150 bp-long DNA fragments upstream of the start codon of *DATIN* (*Rv0079*) were amplified, using DATIN-F and -R primers (Supplementary Table S9), from both *M. tuberculosis* H37Rv and a clinical Mtb strain belonging to sublineage 1.1.1.2 which carried the homoplastic SNP in *Rv0079* intergenic region. The PCR products were purified, cloned into a promoter-less GFP plasmid^59^ and transformed into *E. coli* strain XL1-Gold (Stratagene, USA). A clone harboring the DNA segment was selected and the recombinant plasmid was sequenced to confirm the correct sequence. The plasmid was purified and transformed into *M. smegmatis* mc^2^155 and *M. tuberculosis* H37Ra ATCC 25177 by electroporation. Mycobacterial cells carrying the recombinant plasmid were grown on Middlebrook 7H11 agar plates (Difco, USA) containing 30 µg/ml of kanamycin. GFP expression was measured using a Synergy H1 hybrid Multi-mode microplate reader (Biotek, USA) in the bottom-reading mode. The excitation wavelength was 485 nm and the emission wavelength was 535 nm, with 7H9-OADC as blank. The fluorescence of samples was measured in triplicate wells and repeated three times on different occasions and compared to the wild type strains.

### 5′ Rapid Amplification of cDNA Ends (RACE) assay

Total RNA of the *M. tuberculosis* H37Ra strain was extracted and converted to cDNA. Homopolymeric A-tails were added to the cDNA using the terminal deoxynucleotidyl transferase (TdT) enzyme (Promega, USA). Nested PCR amplification was carried out using gene-specific antisense primers and oligo d(T) anchor primer (Supplementary Table S9). The product of the 5′ RACE reaction was subsequently cloned into pGEM-T Easy vector (Promega, USA) and transformed into *E. coli* XL-1Blue. Finally, DNA sequencing (Bioneer, Korea) using T7 and SP6 universal primers was performed for identification of the 5′ end of the cDNA.

### Statistically analysis

Statistical analyses were performed using SPSS software (version 21.0, SPSS Inc, Chicago, IL). Statistical difference between two groups or among 8 functional groups were evaluated using the nonparametric Mann-Whitney U test and nonparametric Kruskal-Wallis test, respectively. The associations between the homoplastic SNPs and anti-TB resistance, treatment outcomes, AFB smear positivity and HIV status were carried out by Chi-square test. The associations are considered significant at the alpha level of 0.05 with Bonferroni-correction for multiple testing by dividing by the number of homoplastic SNPs (1,229 SNP loci) and 7 tested phenotypes, resulting in the *p*-value threshold of 5.80 × 10^−6^.

## Supplementary information

Supplementary Information.

Supplementary Information2.

Supplementary Information3.

Supplementary Information4.
