# Supplementary material for: Homoplastic single nucleotide polymorphisms contributed to phenotypic diversity in Mycobacterium tuberculosis
Source: Sci Rep. 2020 May 15;10:8024. doi: 10.1038/s41598-020-64895-4 (PMC7229016; doi:10.1038/s41598-020-64895-4)
Supplement: Supplementary file 1 — Supplementary Information. [file 41598_2020_64895_MOESM1_ESM.pdf]

Supplementary information for the manuscript

Homoplastic single nucleotide polymorphisms contributed to phenotypic diversity in *Mycobacterium tuberculosis*

Pornpen Tantivitayakul[1], Wuthiwat Ruangchai[2], Tada Juthayothin[3], Nat Smittipat[3], Areeya Disratthakit[4],

Surakameth Mahasirimongkol[4], Wasna Viratyosin[3], Katsushi Tokunaga[5], Prasit Palittapongarnpim[2 3*]

Description of Supplementary information

Supplementary data:

Predicted functional effects of nonsynonymous SNPs on protein function of Mtb genes related to tuberculosis agent resistance, virulence, cell surface-expose lipid and lipid metabolism, cell wall and cell wall process.

Supplementary Figure S1

Representation of average percentages of the number of homoplastic SNPs per total SNPs identified in each functionally categorized genes of 4 different Mtb lineages: *i)* virulence and detoxification (VF, n=226), *ii)* lipid metabolism (LM, n=238), *iii)* information pathway (IP, n=232), *iv)* cell wall and cell process (CW, n=751), *v) pe/ppe* family protein (PE_PPE, n=168), *vi)* intermediary metabolism and respiration (IMR, n=898), *vii)* regulatory protein (RP, n=193), *viii)* conserved hypothetical protein (CHP, n=1,163). Statistical differences were evaluated with the nonparametric kruskal-wallis test. Asterisks (*) showed the *pe/ppe* groups of Mtb lineage 1, 2 and 3 had higher percentages of homoplastic SNPs per total SNPs than the others with significant difference ($p < 0.05$).

Supplementary Figure S2

The frequency distribution of homoplastic SNPs in coding sequences occurring in 1,170 clinical *M. tuberculosis* isolates.

Supplementary Figure S3

SNP calling workflow performed in this study

Supplementary Figure S4

Position of homoplastic G1340208A SNP (G860A) of *ppe18* in phylogenetic tree of 1,170 *M. tuberculosis* isolates. The phylogeny was reconstructed using Bayesian Interference (BI) methods, which included 4 major lineages (L1: Indo-Oceanic family, L2: East Asian family, L3: East-African Indian family, L4: Euro-American) and 38 sublineages of L1, L2 and L4. Dark blue lines correspond to Mtb isolates carrying homoplastic SNP (G860A). The G860A SNPs were found in all L1 and L2.2 isolates as well as 4 isolates of L4.5.2. Color background shading represents to all Mtb isolates in that sublineages carrying the homoplastic SNPs.

Supplementary Tables S1

Microsoft excel file containing the list of homoplastic SNPs identified in this study. **Sheet1: Table S1.1** corresponds to homoplastic nonsynonymous SNPs found in coding sequences of 8 different functional categories, **Sheet 2: Table S1.2** Homoplastic SNPs in anti-TB resistance genes, **Sheet 3: Table S1.3** Homoplastic SNPs in predicted promoter and **Sheet 4**: **Table S1.4** homoplastic SNPs in intergenic regions of Mtb genes.

Supplementary Tables S2

The number and the density of homoplastic SNPs in *pe/ppe* genes.

Supplementary Table S3

Distribution of homoplastic SNPs in coding regions among 4 different major Mtb lineages**.**

Supplementary Table S4

Distribution of total homoplastic SNPs and homoplastic nonsynonymous SNPs in genes categorized according to essentiality, virulence and antigenicity.

Supplementary Table S5

The homoplastic nsSNPs causing amino acid changes in T cell epitope regions of antigenic proteins.

Supplementary Table S6

Microsoft excel file containing the homoplastic and non-homoplastic SNPs presenting in *ppe18, ppe19, ppe57, ppe59, ppe60* genes as shown in Table S6.1-S6.5, respectively **(Sheet 1-5). Sheet 6**: **Table S6.6**; Representation of average number of total SNPs and homoplastic SNPs in 5 *ppe* genes of 4 Mtb lineages.

Supplementary Table S7

Microsoft excel file containing the SNPs occurring in *ppe18, ppe19, ppe57, ppe59* and *ppe60* identified in 154 complete genomes of *M. tuberculosis* deposited in Genbank database.

Supplementary Table S8

List of top-ranking homoplastic SNPs associated with demographic and clinical characteristics of the patients, including anti-TB drug resistance, Treatment outcomes, HIV status and AFB smear positivity.

Supplementary Table S9

Nucleotide sequences of primers used in the study of the DATIN promoter region.

Supplementary data: Predicted functional effects of nonsynonymous SNPs on protein function

- Genes related to anti-tuberculosis agents

Point mutations in resistance-related genes were primary molecular mechanism of resistance to anti-mycobacterial agents. Among 31 genes related to drug resistance[1-3], 13 genes contained homoplastic SNPs (Supplementary Table S1). Nine homoplastic SNPs in our study were previously identified as first line drug resistance-associated mutations[2,4.] It was consistent with our study population that the strains carrying these SNPs were associated with drug resistance (Supplementary Table S1). These mutations were *katG*315, *inhA*94 and -15C/T *inhA* promoter for isoniazid resistance, *rpoB* codon 445 and 450 for rifampicin resistance, *rpsL* codon 43 and 88 as well as *rrs* A514C and C517T for streptomycin resistance. Besides, we revealed the homoplastic SNPs in genes related to second-line drug resistance; E223K substitution in *ethA* (encodes monooxygenase enzyme which activates prodrug ethionamide); C213R and P156T mutation in *Rv2688c* (encodes ABC Fluoroquinolones efflux pump); P188A substitution in *cycA* (transporter protein responsible for uptake of D-cycloserine).

- Genes related to MTB virulence

The effects of homoplastic nonsynoymous SNPs in genes related to virulence were predicted by all three algorithms (SNAP, polyphen-1, SIFT) as deleterious on protein function, including G96C, T926C in *ppe18* as shown in Supplementary Table S1.

Another notable mutation was E276K in *Rv3283* (*sseA*) encoding probable thiosulfate sulfurtransferase enzyme that involves in anti-oxidative stress mechanism. The E276K mutation was reported to associate with the low abundance of *SseA* protein in modern Beijing B0/W148 strains[5]. These strains showed virulence properties including high transmission rate and multidrug resistance[6,7]. It has been proposed that low sseA protein level might result in the accumulation of reactive oxygen species (ROS)

and lead to induce of DNA mutations resulting in anti-tuberculosis resistance development. Nevertheless we found the E276K mutation in all 235 modern Beijing strains as well as an ancestral Beijing isolate.

- Genes related to cell surface-exposed lipid and lipid metabolism

Cell envelope of MTB is unique and complex, composes of diverse kinds of lipid and glycolipid components. Surface-exposed lipid components of cell envelope include mycolic acids, phthiocerol dimycocerosates (PDIMs), phenolic glycolipids (PGLs), lipomannan (LM), lipoarabinomannan (LAM), sulfolipid-1 (SL-1) and acyltrehalose. These molecules have major roles in host-pathogen interactions during TB infection, such as; entry into macrophage, inhibition of phagolysosome fusion, modulating pro- and anti-inflammatory cytokines[8]. These lipid moieties also serve as antigens that bind to CD1 molecule of antigen presenting cells and then were presented to T lymphocytes[9]. Our investigation found a set of homoplastic SNPs that has high probability to impact on protein function as in the following categories (Supplementary Table S1); *i*) polyketide synthase genes (*pks13* participated in mycolic acid synthesis, *pks5* and *pks12* related to lipooligosaccharides biosynthesis)[10,11], *ii*) papA1 gene encodes acyltransferase enzyme which is essential for sulfolipid-1 biosynthesis[12], *iii*) genes related β-oxidation of fatty acid (*fadB, fadB3, fadE20*). Fatty acids are considered as main carbon source of MTB during infection and latent state. Besides, the β-oxidation of fatty acids plays significant role in formation of cell envelope lipid, especially SL-1, DAT and PAT[13].

- Genes related to cell wall and cell process

We found the homoplastic nsSNPs that have high potential to affect protein function, in the ESX-family proteins (esxH, esxJ, esxL, esxR, esxO, espB and eccB1 and eccA2), the membrane proteins participated in lipid export across cell wall (*mmpL1*, *mmpL9* and *mmpL12*) and lpqW lipoprotein related to biosynthesis of lipoarabinomannan (LAM) in the cell envelope (Supplementary Table S1).

Supplementary References

1 Palomino, J. C. & Martin, A. Drug Resistance Mechanisms in *Mycobacterium tuberculosis*. Antibiotics (Basel) **3**, 317-340 (2014).

2 Roycroft, E. et al. Molecular epidemiology of multi- and extensively-drug-resistant *Mycobacterium tuberculosis* in Ireland, 2001-2014. J Infect **76**, 55-67 (2018).

3 Zhang, Y. The magic bullets and tuberculosis drug targets. Annu Rev Pharmacol Toxicol **45**, 529-564 (2005).

4 Hazbon, M. H. et al. Convergent evolutionary analysis identifies significant mutations in drug resistance targets of *Mycobacterium tuberculosis*. Antimicrob Agents Chemother **52**, 3369-3376 (2008).

5 Bespyatykh, J. et al. Proteome analysis of the *Mycobacterium tuberculosis* Beijing B0/W148 cluster. Sci Rep **6**, 28985 (2016).

6 Mokrousov, I. Insights into the origin, emergence, and current spread of a successful Russian clone of *Mycobacterium tuberculosis*. Clin Microbiol Rev **26**, 342-360 (2013).

7 Lasunskaia, E. et al. Emerging multidrug resistant *Mycobacterium tuberculosis* strains of the Beijing genotype circulating in Russia express a pattern of biological properties associated with enhanced virulence. Microbes Infect **12**, 467-475 (2010)

8. Jackson, M. The mycobacterial cell envelope-lipids. Cold Spring Harb Perspect Med **4**, (2014).

9 Van Rhijn, I. & Moody, D. B. CD1 and mycobacterial lipids activate human T cells. Immunol Rev **264**, 138-153 (2015).

10 Rousseau, C. et al. Virulence attenuation of two Mas-like polyketide synthase mutants of *Mycobacterium tuberculosis*. Microbiology **149**, 1837-1847 (2003).

11 Sirakova, T. D., Dubey, V. S., Kim, H. J., Cynamon, M. H. & Kolattukudy, P. E. The largest open reading frame (*pks12*) in the *Mycobacterium tuberculosis* genome is involved in pathogenesis and dimycocerosyl phthiocerol synthesis. Infect Immun **71**, 3794-3801 (2003).

12 Kumar, P. et al. *PapA1* and *PapA2* are acyltransferases essential for the biosynthesis of the *Mycobacterium tuberculosis* virulence factor sulfolipid-1. Proc Natl Acad Sci U S A **104**, 11221-11226 (2007).

13 Lee, W., VanderVen, B. C., Fahey, R. J. & Russell, D. G. Intracellular *Mycobacterium tuberculosis* exploits host-derived fatty acids to limit metabolic stress. J Biol Chem **288**, 6788-6800 (2013).

Supplementary figures

Figure S1

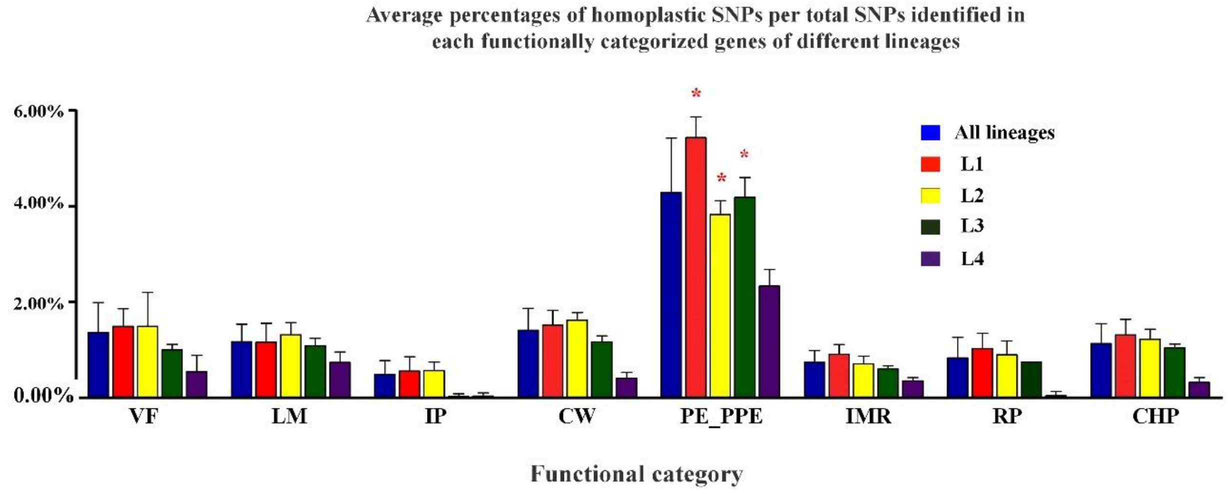

Figure S1 Representation of average percentages of homoplastic SNPs per total SNPs identified in each functionally categorized genes of 4 different Mtb lineages: *i)* virulence and detoxification (VF, n=226), *ii)* lipid metabolism (LM, n=238), *iii)* information pathway (IP, n=232), *iv)* cell wall and cell process (CW, n=751), *v) pe/ppe* family protein (PE_PPE, n=168), *vi)* intermediary metabolism and respiration (IMR, n=898), *vii)* regulatory protein (RP, n=193), *viii)* conserved hypothetical protein (CHP, n=1,163). Statistical differences were evaluated with the nonparametric kruskal-wallis test. Asterisks (*) showed the *pe/ppe* groups of Mtb lineage 1, 2 and 3 had higher percentages of homoplastic SNPs per total SNPs than the others with significant difference ($p < 0.05$).

Figure S2

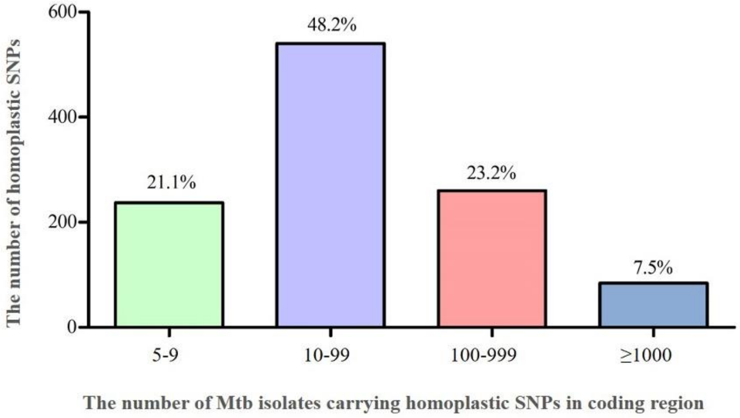

Figure S2 The frequency distribution of homoplastic SNPs in coding sequences occurring in 1,170 clinical *M. tuberculosis* isolates.

Figure S3

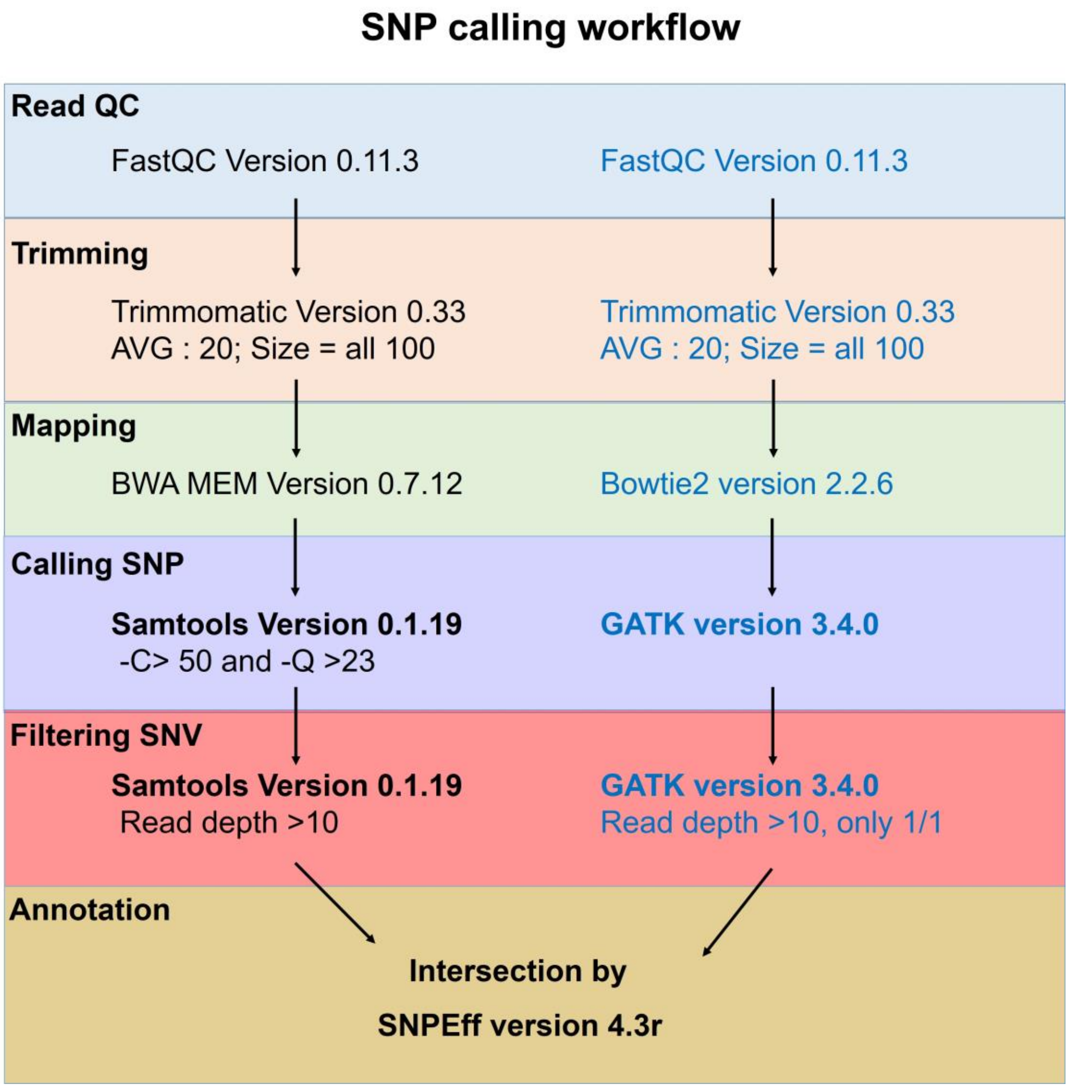

Figure S3 SNP calling workflow performed in this study

Figure S4

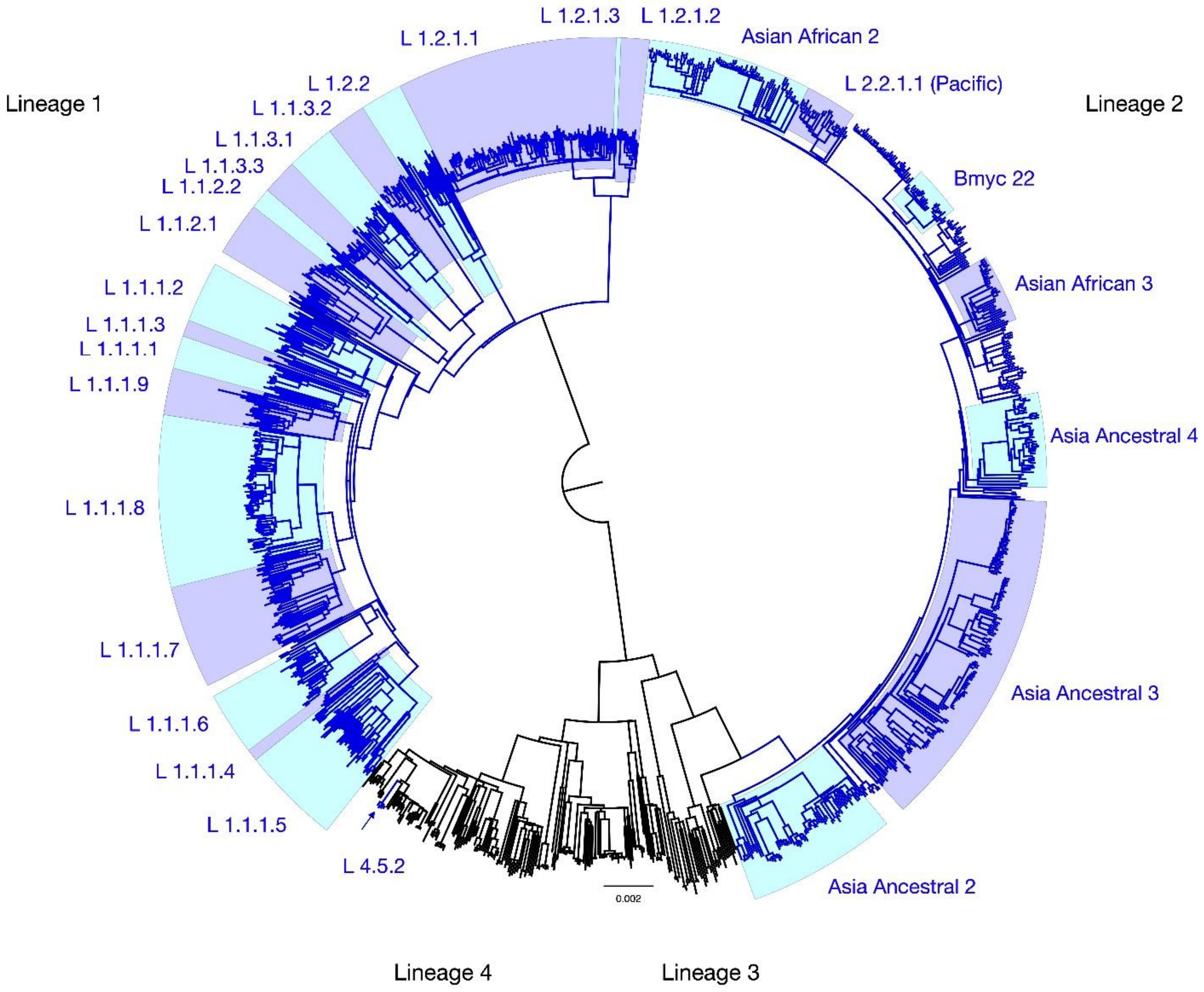

Supplementary Figure S4: Position of homoplastic G1340208A SNP (G860A) of *ppe18* in phylogenetic tree of 1,170 *M. tuberculosis* isolates. The phylogeny was reconstructed using Bayesian Interference (BI) methods, which included 4 major lineages (L1: Indo-Oceanic family, L2: East Asian family, L3: East-African Indian family, L4: Euro-American) and 38 sublineages of L1, L2 and L4. Dark blue lines correspond to Mtb isolates carrying homoplastic SNPs. The G860A SNPs were found in all L1 and L2.2 isolates and 4 isolates of L4.5.2. Color background shading represents to all isolates of that sublineage carrying the homoplastic SNPs.

Supplementary Tables

Supplementary Table S2 The number and the density of homoplastic SNPs in *pe/ppe* genes

Gene (*Rv*)	Gene name	Gene length (bp)	No. of homoplastic sSNPs	No. of homoplastic nsSNPs	Total no. of homoplastic SNPs	Total no. of non-homoplastic SNPs	Total no. of all SNP types	No. homoplastic SNPs per gene length	No. homoplastic nsSNPs per gene length	Ratio of homoplastic SNPs to all SNPs	Ratio of homoplastic nsSNPs to all SNPs
Rv1196	***ppe18***	**1176**	**15**	**22**	**37**	**2**	**39**	**0.031**	**0.019**	**0.949**	**0.564**
Rv3478	***ppe60***	**1182**	**11**	**19**	**30**	**7**	**37**	**0.025**	**0.016**	**0.811**	**0.514**
Rv3429	***ppe59***	**537**	**3**	**9**	**12**	**7**	**19**	**0.022**	**0.017**	**0.632**	**0.474**
Rv1361c	***ppe19***	**1191**	**11**	**7**	**18**	**3**	**21**	**0.015**	**0.006**	**0.857**	**0.333**
Rv3425	***ppe57***	**531**	**3**	**3**	**6**	**0**	**6**	**0.011**	**0.006**	**1.000**	**0.500**
Rv1452c	*pe_pgrs28*	2226	16	6	22	13	35	0.010	0.003	0.629	0.171
Rv0279c	*pe_pgrs4*	2514	12	9	21	11	32	0.008	0.004	0.656	0.281
Rv0980c	*pe_pgrs18*	1374	6	4	10	3	13	0.007	0.003	0.769	0.308
Rv3343c	*ppe54*	7572	25	15	40	17	57	0.005	0.002	0.702	0.263
Rv3018c	*ppe46*	1305	5	1	6	3	9	0.005	0.001	0.667	0.111
Rv3426	*ppe58*	699	0	3	3	0	3	0.004	0.004	1.000	1.000
Rv2591	*pe_pgrs44*	1632	3	3	6	11	17	0.004	0.002	0.353	0.176
Rv1068c	*pe_pgrs20*	1392	4	1	5	5	10	0.004	0.001	0.500	0.100
Rv2107	*pe22*	297	1	0	1	0	1	0.003	0.000	1.000	0.000
Rv1788	*pe18*	300	0	1	1	1	2	0.003	0.003	0.500	0.500
Rv3872	*pe35*	300	0	1	1	2	3	0.003	0.003	0.333	0.333
Rv3022A	*pe29*	315	0	1	1	1	2	0.003	0.003	0.500	0.500
Rv0278c	*pe_pgrs3*	2874	6	3	9	13	22	0.003	0.001	0.409	0.136
Rv0978c	*pe_pgrs17*	996	2	1	3	2	5	0.003	0.001	0.600	0.200
Rv1450c	*pe_pgrs27*	3990	8	4	12	14	26	0.003	0.001	0.462	0.154
Rv0532	*pe_pgrs6*	1785	3	2	5	7	12	0.003	0.001	0.417	0.167
Rv1787	*ppe25*	1098	1	2	3	4	7	0.003	0.002	0.429	0.286
Rv3514	*pe_pgrs57*	4470	5	7	12	12	24	0.003	0.002	0.500	0.292

Gene (*Rv*)	Gene name	Gene length (bp)	No. of homoplastic sSNPs	No. of homoplastic nsSNPs	Total no. of homoplastic SNPs	Total no. of non-homoplastic SNPs	Total no. of all SNP types	No. homoplastic SNPs per gene length	No. homoplastic nsSNPs per gene length	Ratio of homoplastic SNPs to all SNPs	Ratio of homoplastic nsSNPs to all SNPs
Rv0746	*pe_pgrs9*	2352	2	4	6	4	10	0.003	0.002	0.600	0.400
Rv1067c	*pe_pgrs19*	2004	2	3	5	6	11	0.002	0.001	0.455	0.273
Rv3347c	*ppe55*	9474	10	13	23	35	58	0.002	0.001	0.397	0.224
Rv2769c	*pe27*	828	0	2	2	2	4	0.002	0.002	0.500	0.500
Rv3511	*pe_pgrs55*	2145	2	3	5	12	17	0.002	0.001	0.294	0.176
Rv2615c	*pe_pgrs45*	1386	2	1	3	8	11	0.002	0.001	0.273	0.091
Rv0742	*pe_pgrs8*	528	1	0	1	2	3	0.002	0.000	0.333	0.000
Rv2353c	*ppe39*	1065	1	1	2	1	3	0.002	0.001	0.667	0.333
Rv2162c	*pe_pgrs38*	1599	3	0	3	8	11	0.002	0.000	0.273	0.000
Rv0280	*ppe3*	1611	0	3	3	3	6	0.002	0.002	0.500	0.500
Rv3508	*pe_pgrs54*	5706	3	7	10	16	26	0.002	0.001	0.385	0.269
Rv3507	*pe_pgrs53*	4146	3	4	7	17	24	0.002	0.001	0.292	0.167
Rv0305c	*ppe6*	2892	2	2	4	11	15	0.001	0.001	0.267	0.133
Rv0124	*pe_pgrs2*	1464	1	1	2	4	6	0.001	0.001	0.333	0.167
Rv3350c	*ppe56*	11151	7	8	15	55	70	0.001	0.001	0.214	0.114
Rv3345c	*pe_pgrs50*	4617	3	3	6	21	27	0.001	0.001	0.222	0.111
Rv2741	*pe_pgrs47*	1578	1	1	2	9	11	0.001	0.001	0.182	0.091
Rv1753c	*ppe24*	3162	3	1	4	9	13	0.001	0.000	0.308	0.077
Rv1091	*pe_pgrs22*	2562	1	2	3	14	17	0.001	0.001	0.176	0.118
Rv0754	*pe_pgrs11*	1755	2	0	2	5	7	0.001	0.000	0.286	0.000
Rv1803c	*pe_pgrs32*	1920	1	1	2	6	8	0.001	0.001	0.250	0.125
Rv0578c	*pe_pgrs7*	3921	1	3	4	16	20	0.001	0.001	0.200	0.150
Rv1790	*ppe27*	1053	1	0	1	1	2	0.001	0.000	0.500	0.000
Rv3388	*pe_pgrs52*	2196	1	1	2	7	9	0.001	0.000	0.222	0.111
Rv2328	*pe23*	1149	0	1	1	3	4	0.001	0.001	0.250	0.250
Rv2770c	*ppe44*	1149	0	1	1	5	6	0.001	0.001	0.167	0.167
Rv1087	*pe_pgrs21*	2304	1	1	2	10	12	0.001	0.000	0.167	0.083
Rv1706c	*ppe23*	1185	0	1	1	1	2	0.001	0.001	0.500	0.500

Gene (*Rv*)	Gene name	Gene length (bp)	No. of homoplastic sSNPs	No. of homoplastic nsSNPs	Total no. of homoplastic SNPs	Total no. of non-homoplastic SNPs	Total no. of all SNP types	No. homoplastic SNPs per gene length	No. homoplastic nsSNPs per gene length	Ratio of homoplastic SNPs to all SNPs	Ratio of homoplastic nsSNPs to all SNPs
Rv2892c	*ppe45*	1227	0	1	1	0	1	0.001	0.001	1.000	1.000
Rv2340c	*pe_pgrs39*	1242	1	0	1	3	4	0.001	0.000	0.250	0.000
Rv3621c	*ppe65*	1242	1	0	1	5	6	0.001	0.000	0.167	0.000
Rv3595c	*pe_pgrs59*	1320	1	0	1	5	6	0.001	0.000	0.167	0.000
Rv0834c	*pe_pgrs14*	2649	1	1	2	10	12	0.001	0.000	0.167	0.083
Rv0878c	*ppe13*	1332	0	1	1	4	5	0.001	0.001	0.200	0.200
Rv1802	*ppe30*	1392	0	1	1	5	6	0.001	0.001	0.167	0.167
Rv3344c	*pe_pgrs49*	1455	0	1	1	4	5	0.001	0.001	0.200	0.200
Rv1917c	*ppe34*	4380	1	2	3	11	14	0.001	0.000	0.214	0.143
Rv1441c	*pe_pgrs26*	1476	0	1	1	1	2	0.001	0.001	0.500	0.500
Rv0109	*pe_pgrs1*	1491	0	1	1	6	7	0.001	0.001	0.143	0.143
Rv1818c	*pe_pgrs33*	1497	1	0	1	7	8	0.001	0.001	0.125	0.000
Rv3812	*pe_pgrs62*	1515	0	1	1	7	8	0.001	0.001	0.125	0.125
Rv0152c	*pe2*	1578	1	0	1	7	8	0.001	0.000	0.125	0.000
Rv1983	*pe_pgrs35*	1677	0	1	1	4	5	0.001	0.001	0.200	0.200
Rv3159c	*ppe53*	1773	0	1	1	8	9	0.001	0.001	0.111	0.111
Rv0297	*pe_pgrs5*	1776	1	0	1	7	8	0.001	0.000	0.125	0.000
Rv1325c	*pe_pgrs24*	1812	0	1	1	5	6	0.001	0.001	0.167	0.167
Rv2853	*pe_pgrs48*	1848	0	1	1	12	13	0.001	0.001	0.077	0.077
Rv1768	*pe_pgrs31*	1857	1	0	1	7	8	0.001	0.000	0.125	0.000
Rv0755c	*ppe12*	1938	1	0	1	7	8	0.001	0.000	0.125	0.000
Rv0355c	*ppe8*	9903	2	3	5	49	54	0.001	0.000	0.093	0.056
Rv2634c	*pe_pgrs46*	2337	0	1	1	16	17	0.000	0.000	0.059	0.059
Rv2490c	*pe_pgrs43*	4983	1	1	2	17	19	0.000	0.000	0.105	0.053
Rv1759c	*wag22*	2745	1	0	1	6	7	0.000	0.000	0.143	0.000
Rv1651c	*pe_pgrs30*	3036	1	0	1	13	14	0.000	0.000	0.071	0.000
Rv0304c	*ppe5*	6615	0	1	1	27	28	0.000	0.000	0.036	0.036

Supplementary Table S3: Distribution of homoplastic SNPs in coding regions among 4 different major Mtb lineages

**Lineage (L)**	**No. of homoplastic SNPs**	**% of homoplastic SNPs**
All lineages	115	10.3
Within L1	310	27.7
Within L2	119	10.6
Within L3	3	0.3
Within L4	20	1.8
L1&L2	142	12.7
L1&L3	25	2.2
L1&L4	126	11.2
L2&L3	10	0.9
L2&L4	59	5.3
L3&L4	6	0.5
L1&2&3	56	5.0
L1&2&4	113	10.0
L1&3&4	6	0.5
L2&3&4	11	1.0
Total	1,121	100

Supplementary Table S4: Distribution of total homoplastic SNPs and homoplastic non-synonymous SNPs in genes categorized according to essentiality, virulence and antigenicity

Functional Category (No. of genes)		No. of genes carrying homoplastic SNPs (%)	No. of homoplastic SNPs in coding sequences	No. of homoplastic		Ratio of nsSNP/sSNP	Total homoplastic SNP density (per kb)		nsSNP density (per kb)	
				nsSNP	sSNP		Median	Min-Max	Median	Min-Max
**Essentiality***	Essential genes (737)	91 (12.4%)	154	79	75	1.07	0.81	0.19-7.32	0.40	0.00-5.50
	Non-essential genes (3,132)	498 (15.9%)	967	549	418	1.31	1.052	0.15-59.65	0.68	0.00-38.60
**Virulence**	Virulence genes (399)	61 (15.3%)	139	77	62	1.24	0.79	0.16-31.46	0.57	0.00-18.71
	Non-virulence genes (3,470)	528 (15.2%)	982	551	431	1.28	1.00	0.15-59.65	0.65	0.00-38.60
**Antigenicity**	Antigens (411)	112 (27.3%)	446	226	220	1.03	0.90	0.15-59.65	0.65	0.00-38.60
	Non antigens (3,458)	477 (13.8%)	675	402	273	1.47	0.99	0.15-38.60	0.65	0.00-17.54

Total SNP and nsSNP density (per kb) represent total number of homoplastic SNPs and nsSNPs occurring in each gene category divided by the length of genes carrying SNPs in that category.

* Total SNP and nsSNP density (per kb) of non-essential group were higher than that of essential group with statistical difference (p-value < 0.05).

Supplementary Table S5: The homoplastic nsSNPs causing amino acid changes in T cell epitope regions of antigenic proteins.

Gene (*Rv*)	Gene name	IEDB_ID	T cell epitope sequence	START	END	Nucleotide change	Amino acid change
Rv0010c	*Rv0010c*	499711	HSNIKIIRIDEFRR**Y**G	81	96	A284G	Tyr95Cys
Rv0288	*esxH*	42638	MSQIMYNYP**A**MLGHAGDM	1	18	C29T	Ala10Val
		226876	MSQIMYNYP**A**MLGHAGDMAG	1	20		
		501191	MSQIMYNYP**A**MLGHA	1	15		
		103275	IMYNYP**A**MLGHAGDM	4	18		
		738161	IMYNYP**A**ML	4	12		
Rv0928	*pstS3*	40438	LVLDTDS**F**YRPKRPGSYPIV	62	76	T895G	Phe299Val
Rv1037c	*esxI*	145753	DVDAHGAMIRA**Q**AG**S**LEAEH	9	28	A59T, C68T	Gln20Leu, Ser23Leu
		497804	DAHGAMIRA**Q**AG**S**LE	11	25		
		41767	MIRA**Q**AG**S**L	16	24		
Rv1038c	*esxJ*	146003	SGAGWSGMAEATSLDTM**T**QM	41	60	A172G	Thr58Ala
		161694	SGMAEATSLDTM**T**QM	46	60		
		161601	ATSLDTM**T**QMNQAFR	51	65		
		161712	TM**T**QMNQAFRNIVNM	56	70		
Rv1091	*pe_pgrs22*	178885	GAYAAAEAANVSAA**Q**	85	99	G297C	Gln99His
Rv1196	*ppe18*	499426	GSASLVAAA**Q**M**W**DSV	21	35	C88A, G96C	Gln30Lys, Trp32Cys
		503240	VAAA**Q**M**W**DSVASDLF	26	40		
		498971	FQSVVWGLT**V**GSWIG	46	60	G163A, T164C	Val55Met, Val55Ala
		503745	WGLT**V**GSWIGSSAGL	51	65		
		499467	GSWIGSSAGLM**V**AAA	56	70	T200C	Val67Ala
		236357	TVPPPVIAENRAELMIL**I**AT	105	124	T365C	Ile122Thr
		103503	PVIAENRAELMIL**I**A	109	123		
		103365	LMIL**I**ATNLLGQNTP	118	132		

Gene (*Rv*)	Gene name	IEDB_ID	T cell epitope sequence	START	END	Nucleotide change	Amino acid change
Rv1196	*ppe18*	232314	SSKLGGLW**K**	221	229	A686C	Lys229Thr
Rv1196	*ppe18*	179457	**R**SP**I**SNMVSMANNH**M**	235	249	G704T, A712C, A745G	Arg235Leu, Ile238Leu, Met249Val
Rv1196	*ppe18*	103007	AAQAVQTAAQNGV**R**A	274	288	G860A	Arg287Gln
Rv1198	*esxL*	42814	MTINYQFGDVD**A**HGA	1	15	C35A, A59T, G65C, T68C	Ala12Asp, Gln20Leu, Gly22Ala,Leu23Ser
Rv1198	*esxL*	50756	QFGDVD**A**HGAMIRA**Q**	6	20	C35A, A59T, G65C, T68C	Ala12Asp, Gln20Leu, Gly22Ala,Leu23Ser
Rv1198	*esxL*	7529	D**A**HGAMIRALA**GL**LE	11	25	C35A, A59T, G65C, T68C	Ala12Asp, Gln20Leu, Gly22Ala,Leu23Ser
Rv1198	*esxL*	966	AEHQAI**I**SDVL**T**A**S**D	26	40	A94G, C97T, G98C, A109G, A115G	Ile32Val, Arg33Cys, Arg33 Pro, Thr37Ala, Ser39Gly
Rv1198	*esxL*	967	AEHQAIV**R**DVLAAGD	26	40	A94G, C97T, G98C, A109G, A115G	Ile32Val, Arg33Cys, Arg33 Pro, Thr37Ala, Ser39Gly
Rv1198	*esxL*	225572	VLTASDFWGGAGS**A**ACQGFITQ **L**GR	35	59	C143T, T169G	Ala 48Val, Leu57Val
Rv1316c	*ppe19*	118849	INSARMYAGPGSASLVAAA**K**	11	30	A88C, G96C	Lys30Gln, Trp32Cys
Rv1316c	*ppe19*	118825	GSASLVAAA**K**M**W**DSVASDLF	21	40	A88C, G96C	Lys30Gln, Trp32Cys
Rv1316c	*ppe19*	118934	M**W**DSVASDLFSAASAFQSVV	31	50	A88C, G96C	Lys30Gln, Trp32Cys
Rv1316c	*ppe19*	119007	SAASAFQSVVWGLT**T**GSWIG	41	60	A163G, C164T	Thr55Met, Thr55Ala
Rv1316c	*ppe19*	119050	WGLT**T**GSWIGSSAGLMVAAA	51	70	A163G, C164T	Thr55Met, Thr55Ala
Rv1316c	*ppe19*	119020	SSAGLM**V**AAASPYVAWMSVT	61	80	T200C	Val67Ala
Rv1316c	*ppe19*	501193	M**V**AAASPYVAWMSVT	66	80	T200C	Val67Ala
Rv1316c	*ppe19*	119033	TLHSMLKGFAPAAAQAV**E**TA	261	280	G832C	Glu278Gln
Rv1316c	*ppe19*	118900	LSVP**Q**AWAAANQAVTPAARA	321	340	A974C	Gln325Pro
Rv1787	*ppe25*	168525	**F**ATGMAQFFASIAQQ	201	215	C708G	Phe236Leu
Rv1788	*pe18*	169831	TGVVPAAADEV**S**ALT	37	51	C143G	Ser48Trp
Rv1979c	*Rv1979c*	21773	GPRT**R**GYAI	3	11	A21C	Arg7Ser
Rv2770c	*ppe44*	100119	HQAAAVGQAGASA**F**ARQVGL	181	200	T581C	Phe194Ser
Rv2770c	*ppe44*	99903	ASA**F**ARQVGLSHLISDVADA	191	210	T581C	Phe194Ser
Rv2875	*mpt70*	73311	YAAANPTGPASVQGMSQ**D**PV	41	60	G172C	Asp58His
Rv2875	*mpt70*	49635	PTGPASVQGMSQ**D**PVAVAASN NPEL	46	70	G172C	Asp58His

Gene (*Rv*)	Gene name	IEDB_ID	T cell epitope sequence	START	END	Nucleotide change	Amino acid change
Rv3019c	*esxR*	57048	AWQGDTGITYQGWQTQ**W**NQ	41	60	G174T	Trp58Cys
		72986	WQGDTGITYQGWQTQ**W**	43	58		
		106355	DTGITYQGWQTQ**W**NQ	46	60		
		29176	ITYQGWQTQ**W**NQALED	49	64		
		107025	YQGWQTQ**W**NQALEDL	51	65		
		52605	QTQ**W**NQALEDLVRAYQ	55	70		
Rv3347c	*ppe55*	103038	A**L**MSGN**F**SNGILWRG	997	1011	C2992T, T3007G	Leu998Phe, Phe1003Val
Rv3407	*vapB47*	110831	EPARG**R**KRTLSDVLN	79	93	C250T	Arg84Cys
		111006	RG**R**KRTLSDVLNEMR	82	96		
Rv3425	*ppe57*	179778	EEIAANREERRRLIASNVAGVN **T**PA	106	130	A382G	Thr128Ala
		179909	SNVAGVN**T**PAIADLDAQYDQY RARN	121	145		
		179767	AQYDQYRA**R**NVAVMNAYVSW TRSAL	136	160	G431A	Arg144His
		179772	AYVSWTRSALSDL**P**RWREPPQI YRGG	151	176	T491C	Pro164Leu
Rv3478	*ppe60*	179138	LGGLWTAVSPH**L**SP**L**	224	238	T704G, C712A	Leu235Arg, Leu238Ile
		178559	AQNGV**W**AMSSLGSSL	281	295	T856C, T857A	Trp286Arg, Trp286*
Rv3619c	*esxV*	50756	QFGDVDAHGAMIRA**Q**	6	20	C68T	Gln20Leu
		103170	FGDVDAHGAMIRA**Q**A	7	21		
		7530	DAHGAMIRA**Q**AA**S**LE	11	25	A59T	Ser23Leu
Rv3620c	*esxW*	146003	SGAGWSGMAEATSLDTM**T**QM	41	60	A172G	Thr58Ala
		161694	SGMAEATSLDTM**T**QM	46	60		
		161601	ATSLDTM**T**QMNQAFR	51	65		
		161712	TM**T**QMNQAFRNIVNM	56	70		
Rv3883c	*mycP1*	120408	APYNVRR**L**PPPVVEP	398	412	T1214G	Leu405Arg
Rv3887c	*eccD2*	597813	KRWQTAVVTAVVTVCGILA**A**	245	264	G790A	Ala264Thr

* represented premature stop codon

Supplementary Table S8: List of top-ranking homoplastic SNPs associated with demographic and clinical characteristics of the patients, including anti-TB drug resistance, Treatment outcomes, HIV status, and AFB smear positivity.

Table S8.1 List of homoplastic SNPs associated with anti-TB resistance

Anti-TB drug	Gene (Rv)	Gene name	SNP position	Genotype	Amino acid	Total no. isolates	No. of isolates		Pearson Chi-square statistic Value (*p*-value)*
							resistance	sensitive	
Isonazid	*Rv1908c*	*katG*	2155168	wild type G	Ser	1,007	65 (6.5%)	942 (93.5%)	545.204 ($p = 1.00X10^{-13}$)
				G944C	Ser315Thr	85	82 (96.5%)	3 (3.5%)	
	Rv0341	*inhA*	1674481	wild type T	Ser	1,086	141 (13%)	945 (87%)	38.749 ($p = 5.44X10^{-6}$)
				T280G	Ser94Ala	6	6 (100%)	0	
	Rv0341	*inhA*	1673425	wild type C		1,059	118 (11%)	941 (89%)	161.617 ($p = 1.00X10^{-13}$)
				-15C/T		33	29 (88%)	4 (12%)	
Rifampicin	*Rv0667*	*rpoB*	761139	wild type C	His	1,076	35 (3%)	1041 (97%)	345.388 ($p = 1.00X10^{-13}$)
				C1333T	His445Tyr	17	17 (100%)	0	
	Rv0667	*rpoB*	761155	wild type C	Ser	1,078	38 (3.5%)	1040 (96.5%)	263.094 ($p = 1.00X10^{-13}$)
				C1349T	Ser450Leu	15	14 (93%)	1 (7%)	
Ethambutol	*Rv3795*	*embB*	4247429	wild type A	Met	1,083	13 (1%)	1070 (99%)	145.547 ($p = 1.59X10^{-7}$)
				A916G	Met306Val	10	5 (50%)	5 (50%)	
Streptomycin	*Rv0682*	*rpsL*	781822	wild type A	Lys	1,073	87 (8%)	986 (92%)	103.735 ($p = 1.48X10^{-12}$)
				A263G	Lys88Arg	20	15 (75%)	5 (25%)	
	Rv0682	*rpsL*	781687	wild type A	Lys	1,042	55 (5%)	987 (95%)	433.323 ($p = 1.00X10^{-13}$)
				A128G	Lys43Arg	51	47 (92%)	4 (8%)	
	Rv0578c	*pe_pgrs7*	673564	wild type G	Ala	893	53 (6%)	840 (94%)	66.56 ($p = 4.20X10^{-13}$)
				G2353A	Ala785Thr	200	49 (25%)	151 (75%)	
	Rv2471	*aglA*	2774555	wild type T	Val	875	50 (6%)	825 (94%)	67.863 ($p = 2.15X10^{-13}$)
				T992C	Val331Ala	218	52 (24%)	166 (76%)	
	Rv3283	*sseA*	3665753	wild type G	Glu	865	50 (6%)	815 (94%)	61.824 ($p = 1.01X10^{-12}$)
				G826A	Glu276Lys	228	52 (23%)	176 (77%)	

***Bonferroni threshold is $5.80X10^{-6}$**

Table S8.2 List of homoplastic SNPs associated with Treatment outcome

Gene (Rv)	Gene name	SNP position	Genotype	Amino acid	Total no. isolates	No. of isolates associated with outcome of treatment			Pearson Chi-square statistic value (*p*-value)*
						Cure	Failure	Death	
Rv2346c	*esxO*	2626105	wild type T	Leu	646	559 (86.5%)	33 (5.1%)	54 (8.4%)	32.883 (p = 1.11X10^{-7})
			T68C	Leu23Ser	295	219 (74.2%)	12 (4.1%)	64 (21.7%)	
Rv2346c	*esxO*	2626108	wild type G	Gly	656	567 (86.4%	33 (5%)	56 (8.5%)	31.654 (p = 2.10X10^{-7})
			G65C	Gly22Ala	285	211 (74%)	12 (4.2%)	62 21.8%)	
Rv0749	*vapC31*	841495	wild type A	Met	559	489 (87.5%)	27 (4.8%)	43 (7.7%)	29.647 (p = 2.96 X10^{-7})
			A268G	Met90Val	382	289 (75.7%)	18 (4.7%)	75 (19.6%)	
Rv3111	*moaC1*	3479561	wild type G	Asp	559	488 (87.3%)	27 (4.8%)	44 (7.9%)	27.497 (p = 7.86X10^{-7})
			G391A	Asp131Asn	382	290 (75.9%)	18 (4.7%)	74 (19.4%)	
Rv1650	*pheT*	1859989	wild type C	Arg	558	487 (87.3%)	27 (4.8%)	44 (7.9%)	27.201 (p = 9.17X10^{-7})
			C232T	Arg78Trp	383	291 (76%)	18 (4.7%)	74 (19.3%)	
Rv0578c	*pe_pgrs7*	673344	wild type T	Ile	562	490 (87%)	27 (5%)	45 (8%)	26.297 (p = 1.46X10^{-6})
			T2753A	Ile858Asn	379	288 (76%)	18 (5%)	73 (19%)	
Rv1196	*ppe18*	1340052	wild type G	Arg	614	534 (87%)	27 (4%)	53 (9%)	26.003 (p = 2.19X10^{-6})
			G704T	Arg235Leu	327	244 (74.6%)	18 (6%)	65 (20%)	
Rv2955c	*Rv2955c*	3308446	wild type A	Thr	560	488 (87%)	27 (5%)	45 (8%)	25.715 (p = 2.00X10^{-6})
			A100G	Thr34Ala	381	290 (76%)	18 (5%)	73 (19%)	
Rv3429	*ppe59*	3847351	wild type A	Met	560	488 (87%)	27 (5%)	45 (8%)	25.715 (p = 2.00X10^{-6})
			A187T	Met63Leu	381	290 (76%)	18 (5%)	73 (19%)	
Rv0193c	*Rv0193c*	225668	wild type G	Gly	559	487 (87%)	27 (5%)	45 (8%)	25.428 (p = 2.27X10^{-6})
			G904A	Gly302Ser	382	291 (76%)	18 (5%)	73 (19%)	
Rv1262c	*Rv1262c*	1410062	wild type C	Pro	559	487 (87%)	27 (5%)	45 (8%)	25.428 (p = 2.27X10^{-6})
			C311G	Pro104Arg	382	291 (76%)	18 (5%)	73 (19%)	
Rv1409	*ribG*	1585283	wild type A	Lys	559	487 (87%)	27 (5%)	45 (8%)	25.428 (p = 2.27X10^{-6})
			A90C	Lys30Asn	382	291 (76%)	18 (5%)	73 (19%)	
Rv3347c	*ppe55*	3745483	wild type G	Val	559	487 (87%)	27 (5%)	45 (8%)	25.428 (p = 2.27X10^{-6})
			G7702T	Val2568Leu	382	291 (76%)	18 (5%)	73 (19%)	
Rv3507	*pe_pgrs53*	3928892	wild type A	Asp	559	487 (87%)	27 (5%)	45 (8%)	25.428 (p = 2.27X10^{-6})
			A2324G	Asp775Gly	382	291 (76%)	18 (5%)	73 (19%)	
Rv1325c	*pe_pgrs24*	1488645	wild type G	Gly	579	504 (87%)	27 (5%)	48 (8%)	25.195 (p = 2.69X10^{-6})
			G1321C	Gly441Arg	362	274 (76%)	18 (5%)	70 (19%)	
Rv3581c	*ispF*	4024079	wild type A	Gln	558	486 (87%)	27 (5%)	45 (8%)	25.144 (p = 2.65X10^{-6})
			A269G	Gln90Arg	383	292 (76%)	18 (5%)	73 (19%)	

***Bonferroni threshold is 5.80X10^{-6}**

Table S8.3 List of homoplastic SNPs associated with HIV status

Gene (Rv)	Gene name	SNP position	Genotype	Amino acid	Total no. isolates	No. of isolates found in HIV patients	No. of isolates found in non-HIV patients	Pearson Chi-square statistic value (*p*-value)*
Rv3514	*pe_pgrs57*	3948929	wild type G	Ala	899	128 (14%)	771 (84%)	24.140 (p = $6.32X10^{-6}$)
			G3136C	Ala1046Pro	254	71 (28%)	183 (71%)	

***Bonferroni threshold is $5.80X10^{-6}$**

Table S8.4 List of homoplastic SNPs associated with AFB smear positivity

Gene (Rv)	Gene name	SNP position	Genotype	Amino acid	Total no. isolates	No. of isolates found in low grade positive smear	No. of isolates found in high grade positive smear	Pearson Chi-square statistic value (*p*-value)*
Rv1067c	*pe_pgrs19*	1189745	wild type T	Val	1,161	448 (39%)	713 (61%)	4.382 (*p* = 0.048)
			T680C	Val227Ala	7	0	7 (100%)	
Rv3347c	*ppe55*	3750805	wild type T	Phe	1,121	438 (39%)	683 (61%)	6.042 (*p* = 0.014)
			T2380G	Phe794Val	47	10 (21%)	37 (79%)	
Rv3347c	*ppe55*	3750808	wild type G	Asp	1,122	438 (39%)	684 (61%)	5.592 (p = 0.020)
			G2377A	Asp793Asn	46	10 (22%)	36 (78%)	

Low grade refers to scanty or 1+ of AFB smear positivity

High grade refers to 2+ or 3+ of AFB smear positivity

***Bonferroni threshold is $5.80X10^{-6}$**

Supplementary Table S9: Nucleotide sequences of primers used in the study of the *DATIN* promoter region.

Name	Sequence (5` to 3`)
**For amplification of intergenic region of *DATIN***	
Primer DATIN-F	ATT CTA GAG GTG GTG ACA CAG CCC ACA TT
Primer DATIN-R	TAG GAT CCG GCG ACT GCG TTT CGG TTC CA
**5`RACE**	
Primer oligo d(T) adaptor	CCG GAA TTC AAG CTT CTA GAG GAT CCT TTT TTT TTT TTT TTT
Primer PM1 adaptor	CCG GAA TTC AAG CTT CTA GAG GAT CC
Primer cDATIN	CTG GTC GGT GAA AAA CAG GAA TGG
Primer GSPD1	GCA CGA TCT GTC GAT CCA GTC TG
Primer GSPD2	GCG CCC TTA ATG GGG TGT CAC
**For sequencing of cloned fragments**	
Primer PFPV2-F	GAT GTA CGT GGC GAA CTC CG
Primer PFPV2-R	CCT TCA CCC TCT CCA CTG ACA G
